# Recent Foreign Papers

**Published:** 1871-12

**Authors:** 


Recent Foreign Papers.—Professor Stricker's "New Year-Book of Medicine," of which too parts have been published, contains a series of valuable papers on microscopy. Among them may be mentioned the following :—There is one by Dr. Hausen on the results of inflammation in the corneal tissue; another by Dr. Güterbock on the effects of inflammation of tendons; by Dr. Yeo on the pathology of inflamed lymphatic glands; by Stricker himself on the nature of the poison of pus, and another paper in conjunction with Dr. Albert—an account of traumatic fever; Dr. Lang gives the pathology of inflammation of the bones, and Dr. Kimdrat a paper on the inflammatory changes in the endothelia of serous membranes.

